# Combined Inactivation of MYC and K-Ras Oncogenes Reverses Tumorigenesis in Lung Adenocarcinomas and Lymphomas

**DOI:** 10.1371/journal.pone.0002125

**Published:** 2008-05-07

**Authors:** Phuoc T. Tran, Alice C. Fan, Pavan K. Bendapudi, Shan Koh, Kim Komatsubara, Joy Chen, George Horng, David I. Bellovin, Sylvie Giuriato, Craig S. Wang, Jeffrey A. Whitsett, Dean W. Felsher

**Affiliations:** 1 Department of Radiation Oncology, Stanford University School of Medicine, Stanford, California, United States of America; 2 Division of Oncology, Department of Medicine, Stanford University School of Medicine, Stanford, California, United States of America; 3 Division of Oncology, Department of Pathology, Stanford University School of Medicine, Stanford, California, United States of America; 4 Institut National de la Santé et de la Recherche Médicale (INSERM) U563 Centre de physiopathologie Toulouse Purpan, Toulouse, France; 5 Université Paul-Sabatier, Toulouse, France; 6 Division of Pulmonary Biology, Cincinnati Children's Hospital Medical Center and University of Cincinnati College of Medicine, Cincinnati, Ohio, United States of America; Northwestern University, United States of America

## Abstract

**Background:**

Conditional transgenic models have established that tumors require sustained oncogene activation for tumor maintenance, exhibiting the phenomenon known as “oncogene-addiction.” However, most cancers are caused by multiple genetic events making it difficult to determine which oncogenes or combination of oncogenes will be the most effective targets for their treatment.

**Methodology/Principal Findings:**

To examine how the MYC and *K-ras^G12D^* oncogenes cooperate for the initiation and maintenance of tumorigenesis, we generated double conditional transgenic tumor models of lung adenocarcinoma and lymphoma. The ability of MYC and *K-ras^G12D^* to cooperate for tumorigenesis and the ability of the inactivation of these oncogenes to result in tumor regression depended upon the specific tissue context. MYC-, *K-ras^G12D^*- or MYC/*K-ras^G12D^*-induced lymphomas exhibited sustained regression upon the inactivation of either or both oncogenes. However, in marked contrast, MYC-induced lung tumors failed to regress completely upon oncogene inactivation; whereas *K-ras^G12D^*-induced lung tumors regressed completely. Importantly, the combined inactivation of both MYC and *K-ras^G12D^* resulted more frequently in complete lung tumor regression. To account for the different roles of MYC and *K-ras^G12D^* in maintenance of lung tumors, we found that the down-stream mediators of *K-ras^G12D^* signaling, Stat3 and Stat5, are dephosphorylated following conditional *K-ras^G12D^* but not MYC inactivation. In contrast, Stat3 becomes dephosphorylated in lymphoma cells upon inactivation of MYC and/or *K-ras^G12D^*. Interestingly, MYC-induced lung tumors that failed to regress upon MYC inactivation were found to have persistent Stat3 and Stat5 phosphorylation.

**Conclusions/Significance:**

Taken together, our findings point to the importance of the K-Ras and associated down-stream Stat effector pathways in the initiation and maintenance of lymphomas and lung tumors. We suggest that combined targeting of oncogenic pathways is more likely to be effective in the treatment of lung cancers and lymphomas.

## Introduction

Cancer is largely caused by the summation of activated oncogenes and inactivated tumor-suppressors that occur in a permissive epigenetic milieu resulting in various pathologic features: autonomous proliferation, immortalization, blocked differentiation, the induction of angiogenesis, capacity for invasion, resistance to apoptosis and genomic instability [1]. Transgenic mouse models have been a valuable means to identify cooperating oncogenic events relevant to human tumorigenesis. A classic example is the forced coexpression of c-myc and v-Ha*-ras* oncogenes *in vivo* resulting in a strongly synergistic tumorigenesis phenotype [2]. MYC encodes a transcription factor that regulates the expression of a multitude of genes involved in regulating cellular proliferation and growth and when overexpressed results in the prototypical pathologic features of cancer as described above [3], [4]. *K-ras* encodes a low-molecular weight GTP-binding protein responsible for transmitting signals from receptor tyrosine kinases to downstream modulators of cell growth and survival [5], [6] and has been shown to stabilize the MYC protein [7]. Thus, MYC and *ras* cooperate to induce tumorigenesis through multiple mechanisms.

Conditional mouse models allowing temporal control of oncogene expression have become increasingly important for teasing apart the tumorigenesis pathways in adult tissue compartments [8]. Comparison of different transgenic systems would also suggest that tissue type plays a role on the ability of oncogenes to promote tumorigenesis [9], [10]. Conditional transgenic tumor models have permitted the investigation of how oncogenes not only initiate but maintain tumorigenesis in different tissue and developmental contexts. Using these models, it has been established that many experimental mouse tumors exhibit the phenomenon of oncogene addiction [11], [12], whereby the inactivation of a single oncogene has been shown to be sufficient to induce sustained tumor regression [13]–[30]. Human tumors also appear to exhibit oncogene addiction [31]–[33]. Most notably, chronic myelogenous leukemia (CML) [34], [35] and gastrointestinal stromal tumor (GIST) are highly sensitive to treatment with the tyrosine kinase inhibitor, imatinib [36].

Since most human cancers are genetically complex and are associated with the activation of more than one oncogene, strategies targeting multiple oncogenes appear to be a logical approach for the treatment of human cancers [1], [37], [38]. Notably, elegant studies illustrated that breast adenocarcinomas induced by conditional MYC overexpression but that also subsequently develop mutations in *K-Ras* fail to undergo sustained regression upon MYC inactivation [16], [17]. These results suggest that the combined inactivation of both MYC and mutant Ras may be more effective in inducing sustained tumor regression. However, to date it has not been directly examined if the coordinate inactivation of both MYC and mutant Ras would be more effective in inducing sustained tumor regression.

To study how MYC and *K-ras^G12D^* cooperate for the initiation and maintenance of tumorigenesis, we have generated double conditional transgenic mouse models of lymphoma and lung adenocarcinoma. MYC-, *K-ras^G12D^*- or MYC/*K-ras^G12D^*-induced lymphomas exhibited sustained regression upon single or double oncogene inactivation. Interestingly, in contrast to most MYC-induced tumor models, MYC-induced lung tumors were not oncogene-addicted; whereas *K-ras^G12D^* inactivation did induce complete tumor regression in *K-ras^G12D^*–induced lung tumors. Furthermore, the combined inactivation of MYC and *K-ras^G12D^* was associated with reversible lung tumorigenesis. In addition, we observed that down stream K-Ras effector, Stat3, was down-regulated upon oncogene inactivation in lung tumors and lymphomas that regressed. However, non-regressing MYC-induced lung tumors were found to have aberrantly active Stat3 signaling. These data have important implications for treatment strategies where use of multiple targeted agents is being considered and highlight the significance of the K-Ras and Stat pathways for tumorigenesis and tumor maintenance.

## Results

### MYC inactivation alone fails to induce regression of lung cancer

**Figure 1:**
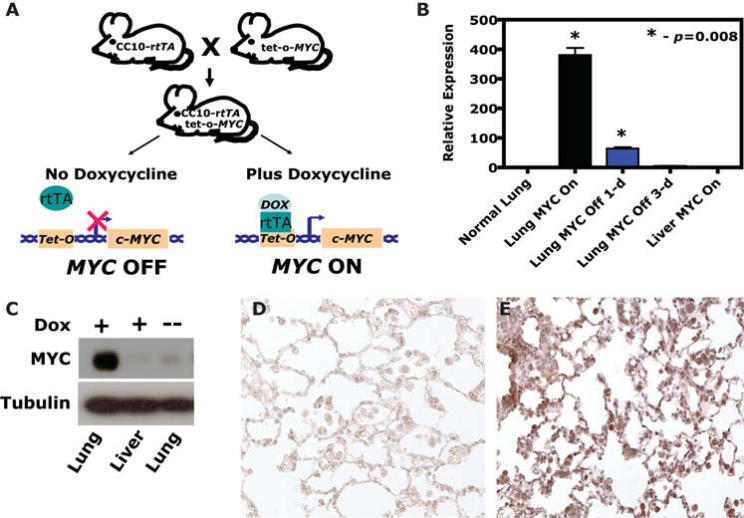
Generation and validation of a murine conditional lung specific MYC model. (A) A mouse line containing the Clara cell secretory protein (CCSP or CC10) promoter driving the reverse tetracycline transactivating protein (rtTA) is crossed with a line containing MYC under the control of the tetracycline-responsive promoter (TetO). In the bitransgenic animal, *CCSP-rtTA*/*TetO-c-MYC* (or CM), absence of doxycycline prevents rtTA protein from binding and activating the *TetO* promoter. Addition of doxycycline triggers a conformational change which enables *TetO* binding, activation and MYC transcription. (B) Quantitative real-time reverse transcriptase-PCR (qRT-PCR) using primers specific for the MYC transgene demonstrate that bitransgenic CM animals have robust conditional and lung restricted expression of MYC (n> = 3; see Methods for specifics). (C) Western blotting of tissue from bitransgenic CM animals using a human specific MYC antibody (9E10) reveals similar robust MYC protein expression in a conditional and lung restricted manner. Immunohistochemical staining with a cross species reacting MYC antibody (C19) revealed similar conditional MYC expression as confirmed by (D) MYC inactivated and (E) MYC activated CM lungs.

To examine the role of MYC in the initiation and maintenance of tumorigenesis, transgenic mice were generated that exhibit conditional expression of the human c-*MYC* oncogene (referred to as MYC from now on) by crossing *TetO-MYC* transgenic mice [15] with the *CCSP-rtTA* transgenic line [39] generating *CCSP-rtTA/TetO- MYC* mice (now termed CM; see Figure 1A). The *CCSP-rtTA* mouse line contains the Clara cell secretory protein (CCSP or CC10) promoter which drives expression of the reverse tetracycline transactivating protein (rtTA) in lung Clara cells, alveolar Type II pneumocytes and some other non-ciliated bronchial and bronchiolar epithelial cells [23], [39]. To verify conditional regulation, CM mice were examined for MYC expression using quantitative real-time polymerase chain reaction (qRT-PCR). The addition of doxycycline induced expression of MYC transcripts 380-fold in the lung with no appreciable expression in non-induced lung tissue or induced liver (Figure 1B). Similar to previous reports using the *CCSP-rtTA* line, the kinetics of inactivation revealed background MYC expression by 3-days after doxycycline withdrawal [23], [39]. Examination for MYC protein revealed similar robust inducible regulation by western blotting (Figure 1C) and immunohistochemical (IHC) analysis in CM lung tissue (Figure 1D–E). Notably, two target genes of MYC, ornithine decarboxylase (ODC) and nucleolin [3], were found to exhibit expression that was coordinately regulated in a conditional manner as expected from a functional MYC protein (Figure S1). Thus, we have developed a conditional model for the expression of MYC in the lung.

**Figure 2:**
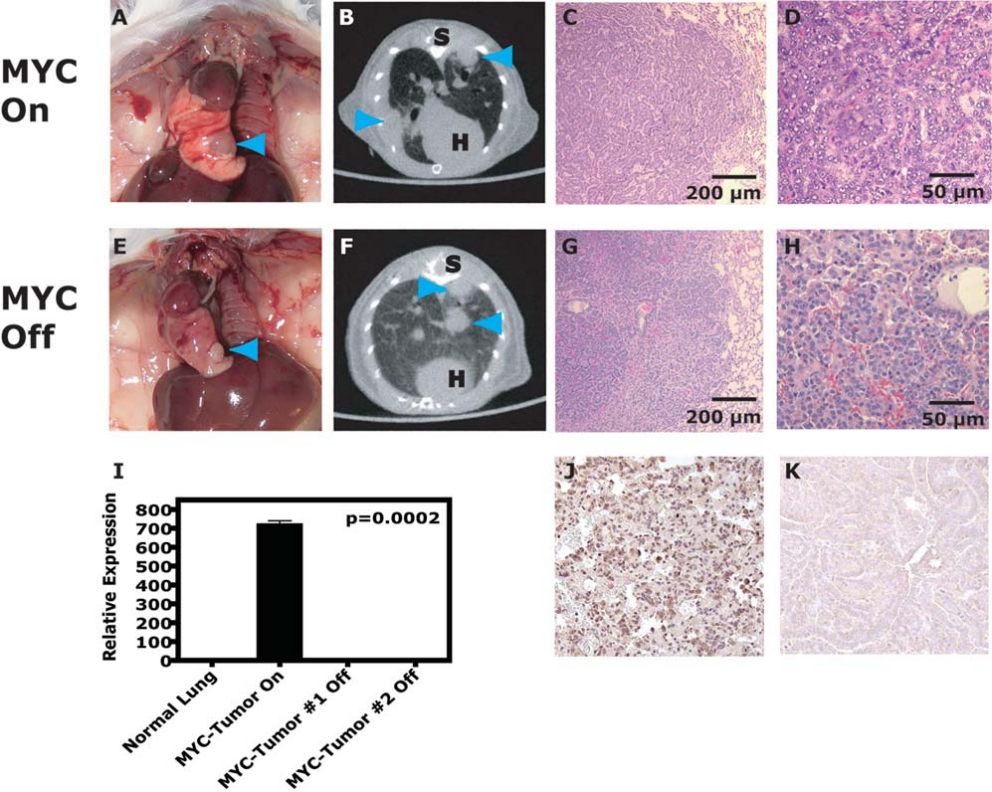
Conditional expression of MYC in the lung predisposes to bronchiogenic adenocarcinomas that are oncogene-independent. (A) MYC expression in the lung results in lung adenocarcinomas as shown (B) radiographically and (C & D) microscopically. MYC-induced tumors after 10 weeks of MYC-inactivation (removal of doxycycline) are still present on (E) gross examination, (F) radiographically and (G & H) microscopically (n> = 10). Necropsy photographs demonstrate the thorax with tumors marked by blue arrowheads (A & E). MicroCT was used to serially monitor the mice and representative axial images are shown with tumors marked by blue arrowheads (B & F); S–spine and H–heart. H&E histology of germane sections show viable adenocarcinoma cells (C, D, G and H). To rule out the possibility that MYC-induced lung tumors had developed doxycycline (or TetO)-dysregulated MYC expression, inactivated MYC-induced lung tumors were examined for spurious expression of MYC at the mRNA and protein level. (I) qRT-PCR analysis of MYC-induced lung tumors from CM mice that had been inactivated for greater than 10 weeks demonstrated no expression of the MYC transgene in contrast to a MYC-induced tumor that had never been inactivated. Representative immunohistochemical analysis (performed like Figure 1D) of an (K) inactivated MYC-induced tumor also showed lack of MYC transgene product and endogenous murine MYC protein compared to (J) a MYC-induced positive tumor control (> = 3-9 tumors for > = 3 mice per experiment).

Induction of MYC in the lung epithelium by the administration of doxycycline in the drinking water of CM mice uniformly resulted in tumorigenesis (Figure 2A) that on histologic examination were consistent with adenomas or adenocarcinomas (Figure 2C–D) [40]. Tumors were composed of cuboidal to columnar cells lining alveoli frequently containing vacuolated tumor cells, multiple nucleoli and mitoses. Using the consensus classification system as developed by Yu and colleagues, these tumors would be classified as adenoma–mixed subtype (1.2.1.2.3) and adenocarcinoma–NOS (1.2.3.2.5) [40]. Activated tumor cells stained intensely for MYC protein by IHC analysis (Figure 2J) and were TTF-1 positive as expected (data not shown). To enhance detection and allow serial monitoring of lung tumors during growth and following interventions in our study, micro-computed tomography (µCT) was performed on cohorts of mice for the detection of millimeter sized lesions (Figure 2B). CM mice developed tumors with a median latency of 52 weeks as detected by µCT screening usually well before clinical signs developed. CM mice usually developed 1–2 dominant tumors that were located more centrally in the mediastinum (Figure 2B). Thus, MYC induction by the *CCSP* promoter is sufficient to induce lung adenocarcinomas.

To simulate MYC targeted treatment and evaluate if MYC inactivation was sufficient to reverse lung tumorigenesis, doxycycline treatment was removed to suppress expression of the transgene. Surprisingly, 98% (n = 51) of tumor bearing CM mice did not exhibit complete tumor regression following doxycycline withdrawal as demonstrated by gross examination on necropsy, radiographically and/or histologically (Figure 2E–H). Only 1 out of 8 CM mice demonstrated volumetric tumor regression greater than 60% by radiographic exam following 6 weeks of doxycycline withdrawal (and see below). A trivial explanation for doxycycline-independent tumor viability could be either the aberrant expression of MYC independent of doxycycline or endogenous upregulation of murine c-Myc. To address this possibility qRT-PCR and IHC were performed on tissue from mice in which MYC was inactivated but the tumors had not regressed. No transgene or protein expression of MYC were detected (Figure 2I–K and data not shown, n = 6). Since the anti-MYC antibody used for IHC in our study also cross reacts with murine c-Myc, we concluded that these tumors also had not upregulated endogenous c-Myc (Figure 2J–K). Taken together these results suggest continuously activated CM mice develop lung tumors that become independent of MYC for tumor maintenance.

### MYC and *K-ras^G12D^* cooperation for tumorigenesis is dependent on tissue type

**Figure 3:**
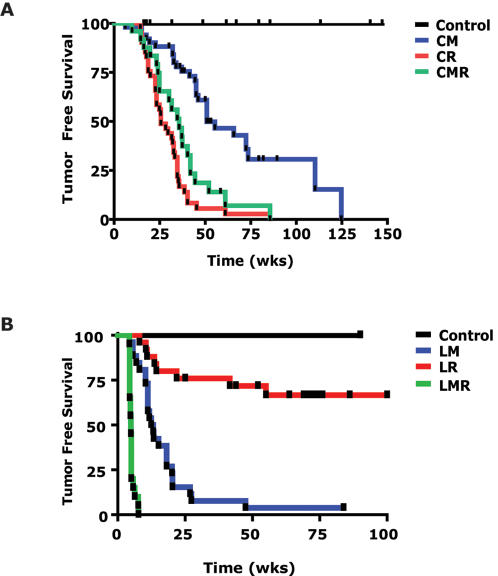
Cooperation during tumorigenesis by conditional MYC and *K-ras^G12D^* oncogenes. (A) Kaplan-Meir analysis of Tumor Free Survival for oncogene-induced lung tumorigenesis. Single MYC- (CM, n = 51) and *K-ras^G12D^* -induced (CR, n = 41) lung tumors arose with a median latency of 52 and 26 weeks, respectively, after conditional oncogene activation. The double conditional oncogene animals (CMR, n = 25) had a median latency that were no different than the single CR animals by log-rank analysis suggesting that *K-ras^G12D^* was epistatic to MYC for lung tumorigenesis. A syngenic control cohort consisting of wildtype mice, those with MYC/*K-ras^G12D^* (without CCSP), CCSP alone, or *K-ras^G12D^* alone were fed water and never developed lung tumors (n = 8). Tumor Free Survival was scored by serial µCT imaging of animals following addition of doxycycline at 3–4 weeks of age. (B) Kaplan-Meir analysis of Tumor Free Survival for oncogene-induced lymphomagenesis. MYC-induced lymphomas (LM, n = 26) arose with a median latency of 13 weeks after conditional oncogene activation. In contrast, less than half of the mice developed lymphoma after 100 weeks of conditional *K-ras^G12D^* activation (LR, n = 25). The double conditional oncogene animals (LMR, n = 22) had a median latency that was significantly different than either single oncogene line (5 weeks, *p*< = 0.0001 by log rank), suggesting that *K-ras^G12D^* and MYC were cooperative for lymphomagenesis. Control animals fed water never developed tumors (n = 6). Tumor Free Survival was scored when animals were moribund with tumor.

**Figure 4:**
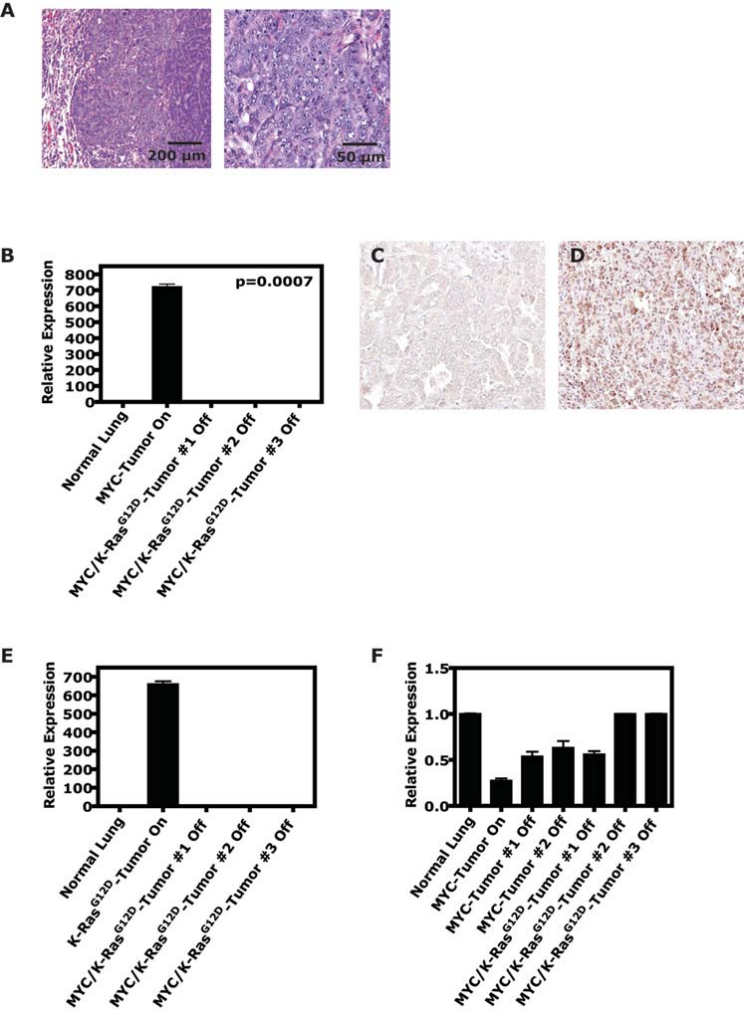
Conditional expression of MYC/*K-ras^G12D^* in the lung predisposes to bronchiogenic adenocarcinomas. (A) Double MYC/*K-ras^G12D^* (CMR)-induced tumors have histology consistent with adenomas/adenocarcinomas similar to MYC- and *K-ras^G12D^*-induced tumors on H&E. (B) To rule out the possibility that the double oncogene-induced lung tumors had developed doxycycline (or TetO)-dysregulated MYC or *K-ras^G12D^* expression, inactivated double oncogene-induced lung tumors were examined for spurious expression of MYC and *K-ras^G12D^* at the mRNA and/or protein level. qRT-PCR analysis of double oncogene-induced lung tumors from CMR mice that had been inactivated (doxycycline removed from drinking water) for 2–9 weeks demonstrated lack of expression of the MYC transgene in contrast to a MYC-induced tumor that had never been inactivated. Immunohistochemical analysis (performed like Figure 1D) on similar (C) inactivated double oncogene-induced tumors also showed lack of MYC transgene product and endogenous murine MYC protein compared to a (D) MYC-activated tumor. qRT-PCR analysis of double oncogene-induced lung tumors from CMR mice that had been inactivated demonstrated no expression of the (E) *K-ras^G12D^* transgene or upregulation of (F) endogenous murine *K-ras* (> = 3–9 tumors for > = 3 mice per experiment).

We were surprised to find that CM tumors were independent of MYC, since a multitude of previous studies have demonstrated that MYC-induced tumors exhibit complete tumor regression upon MYC inactivation [13]–[15], [17], [21], [22]. Studies demonstrated a subset of breast tumors induced by MYC overexpression that fail to undergo sustained regression upon MYC inactivation have mutations in *K-Ras* [16], [17]. To evaluate in our conditional lung model if MYC and *K-Ras* cooperate, we utilized a conditional mutant *K-ras^G12D^* line previously described, *CCSP-rtTA*/*TetO-K-ras^G12D^* (now called CR) [23]. CM and CR were then used to produce the bi-conditional animals *CCSP-rtTA/TetO- MYC/TetO-K-ras^G12D^* (or CMR), which upon doxycycline administration simultaneously overexpress both oncogenes under the control of the CCSP promoter in lung and as described below in lung tumors (Figure S2). At 3–4 weeks of age cohorts of CM, CR and CMR mice were treated with doxycycline and screened using physical exam and µCT screening. As described above, CM mice developed lung tumors with a median latency of 52 weeks (Figure 3A). Upon doxycycline treatment, CR mice developed lung adenocarcinomas with a median latency of 26 weeks. Surprisingly, CMR mice developed lung adenomas and adenocarcinomas (Figure 4A) with a latency of 36 weeks similar to the CR mice (Figure 3A; not significantly different by log-rank analysis, *p*>0.05). Thus, in the setting of adult lung epithelium MYC and *K-ras^G12D^* failed to cooperate to induce accelerated tumorigenesis.

We were surprised that the conditional MYC and *K-ras^G12D^* oncogenes did not cooperate to induce lung tumorigenesis. To evaluate if these transgenes would cooperate in another tissue setting, we induced expression of either oncogene alone or together in lymphocytes utilizing an *Eμ-SR-tTA* line (data not shown). In contrast to what we observed in the lung, we found that MYC (LM) was a much more potent oncogene than *K-ras^G12D^* (LR) at inducing lymphomas with a median latency of tumor onset of 13 weeks *versus* more than 100 weeks (Figure 3B; *p*<0.0001 by log rank). Moreover, we found that MYC and *K-ras^G12D^* cooperate to induce tumorigenesis with a reduced median latency of 5 weeks (all curves different, *p*<0.0001 by log rank). Thus, MYC and *K-ras^G12D^* cooperate to induce lymphoma but not lung adenocarcinoma.

### Combined MYC/*K-ras^G12D^* inactivation induces complete tumor regression

**Figure 5:**
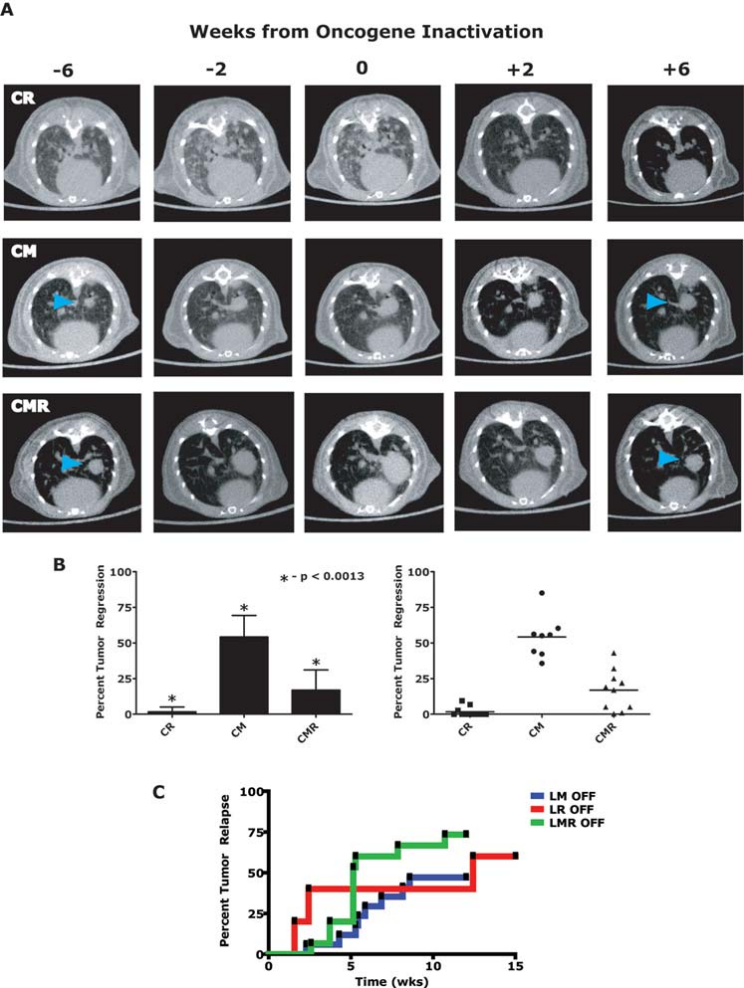
Regression of tumors following dual MYC/*K-ras^G12D^*-oncogene inactivation. (A) Representative serial µCT images of single and double-oncogene-induced lung tumors following withdrawal of doxycycline for 6 weeks. CR (*CCSP-rtTA/TetO-K-ras^G12D^*, n = 11) animals demonstrated rapid tumor regression within ∼2 weeks following inactivation of the oncogene. In contrast, CM (*CCSP-rtTA/TetO-c-MYC*, n = 8) mice did not demonstrate full tumor regression even after 6 weeks following oncogene inactivation. Interestingly, CMR (*CCSP-rtTA/TetO-c-MYC*/*TetO-K-ras^G12D^*, n = 10) animals on a whole exhibited an intermediate level of tumor regression, with some tumors regressing completely, compared to the single oncogene-induced lung tumors. (B) Left panel shows the mean with standard deviation of normalized tumor volumes from (A) at 6 weeks following oncogene-inactivation. The relative genotype order for mean tumor regression: CR (n = 11)>CMR (n = 8)>CM (n = 10); all pair-wise comparisons were p<0.0013. The right panel demonstrates same data as the left panel but in a scatter plot form with the mean denoted as a horizontal line. (C) Kaplan-Meir analysis of tumor-free survival of conditional lymphoma mice. Oncogene inactivation in MYC-induced lymphoma resulted in sustained regression in more than half the mice (LM OFF, n = 15). Oncogene inactivation in mice with *K-ras^G12D^*–induced lymphoma resulted in tumor regression and increased median survival by 12 weeks (LR OFF, n = 5). Inactivating both MYC/*K-ras^G12D^* together also resulted in tumor regression and increased median survival by 5 weeks. There is no significant difference between inactivating MYC/*K-ras^G12D^* together vs *K-ras^G12D^* alone (log rank analysis *p* = 0.4849). Relapse free survival was scored when mice were moribund with tumor burden.

We speculated that in our MYC-induced lung tumors, activation of the Ras signaling pathway may provide a means to bypass the requirement for MYC, as has been previously suggested [16], [17]. To directly test this hypothesis, we simulated double targeted treatment of MYC and *K-ras^G12D^* using dual conditional CMR tumor laden mice by inactivating both oncogenes (Figure 4B–F) and then comparing similarly to the single CM and CR mice. Serial µCT imaging was performed on cohorts of CR, CM and CMR lung tumor bearing mice prior to and following oncogene-inactivation was performed (Figure 5A). CR mouse tumors demonstrated complete lung tumor regression following oncogene inactivation, as has been described previously (n = 11; Figure 5A–B) [23]. In marked contrast, CM tumors failed to regress completely, as described above (n = 8; Figure 5A–B). Tumor bearing CMR mice on the whole exhibited tumor regression intermediate to that of the CR and CM mice following dual oncogene-inactivation of MYC and *K-ras^G12D^* oncogenes (n = 10; Figure 5A–B). However as predicted, 40% of the individual CMR-induced lung tumors analyzed showed complete tumor regression equal to those from CR mice following doxycycline withdrawal (Figure 5B). By qRT-PCR and IHC, we confirmed that MYC, transgenic *K-ras^G12D^* and endogenous *K-Ras* were not expressed in the inactivated CMR lung tumors and CMR tumors were indeed inactivated for both oncogenes (Figure 4B–F). Altogether, these data suggest that activation of the K-Ras signaling pathway is an essential rate-limiting event during lung tumorigenesis. Moreover, our observation that the combined inactivation of both MYC and *K-ras^G12D^* induced lung tumor regression more effectively suggests that the K-Ras pathway may be an important target for the treatment of lung cancer.

From these results, we speculated that *K-Ras* may be mutated in MYC-induced lung tumors, as has been observed in MYC-induced breast tumors [16], [17], [20]. To address this possibility, we sequenced three MYC-inactivated CM tumors (or derived cell lines) for mutations in *K-Ras* but were unable to detect any hotspot activating mutations (Figure S3). Therefore, activating mutations of *K-ras* were not a common occurrence and was not an explanation for MYC independence in our lung model, in contrast to what has been previously reported for breast cancer [16], [17], [20]. Other components of the K-Ras effector pathway were obvious next candidates and were investigated as described below.

Notably, in the lymphoma model, inactivation of MYC (LM Off), *K-ras^G12D^* (LR Off) or MYC/*K-ras^G12D^* together (LMR Off) were each able to induce complete regression of lymphomas and extend tumor free survival (Figure 5C; no difference by log rank analysis, *p*>0.05). For lymphomas, inactivation of MYC or *K-ras^G12D^* alone or both MYC/*K-ras^G12D^* induced reversible tumorigenesis. In contrast, single *K-ras^G12D^* and dual MYC/*K-ras^G12D^* inactivation induced reversible lung tumorigenesis, but MYC inactivation alone failed to reverse tumorigenesis. Thus, whether MYC induces reversible tumorigenesis alone is dependent upon the specific tumor context.

### Tumor regression is associated with the dephosphorylation of Stat3

**Figure 6:**
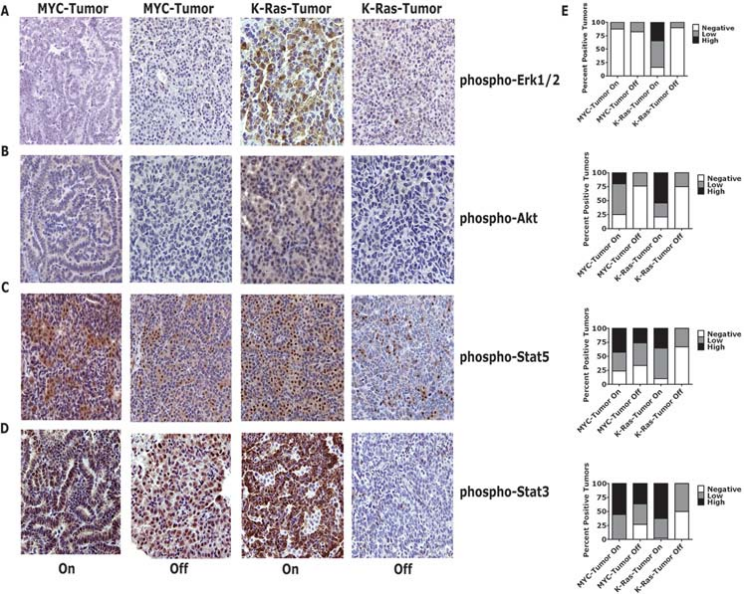
Persistent activation of down-stream Ras signaling pathways after MYC inactivation. (A) Representative MYC-induced lung tumors do not show phospho-Erk1/2 staining by IHC during activation (n = 4) or inactivation (n = 6). (B) Similarly, inactivated MYC-induced lung tumors do not show phospho-Akt staining by IHC (n = 6). Strong conditional staining for both phospho-Erk1/2 and phospho-Akt are seen in activated (or “On”) *K-ras^G12D^*–induced tumors (n = 3) but not inactivated (or “Off”) tumors (n = 7–8). A majority of MYC-induced lung tumors demonstrated (C) phospho-Stat5 (4/6) and/or (D) phospho-Stat3 (6/7) staining by IHC that was independent of doxycycline which was in contrast to *K-ras^G12D^*–induced tumors (n = 3 “On” & 6 “Off”). IHC was performed similar to Figure 1D with stated antibodies with MYC-induced lung tumors that were activated or inactivated (1–10 weeks). (E) IHC staining was scored as negative, low (<50% positive cells) or high (≥50% positive cells) for phospho-Erk1/2, -Akt1, phospho-Stat5 and phospho-Stat3 positive tumors.

Our results suggested that the K-Ras signaling pathway is crucial for both the initiation and maintenance of lung tumorigenesis. Important upstream and downstream regulators of the K-Ras pathway are the epidermal growth factor receptor (EGFR) and Erk1/2, Akt1 and Stat3/5, respectively [27], [41]–[50]. The upstream effector, EGFR, was not found to be phosphorylated in any of our MYC- or *K-ras^G12D^*-induced lung tumors by IHC (data not shown) and as described previously for K-Ras-induced lung tumors [27]. As expected, we observed phosphorylation of Erk1/2 in *K-ras^G12D^*-induced tumors decrease upon *K-ras^G12D^* inactivation (CR; Figure 6A). However, there was no evidence for phosphorylated-Erk1/2 staining by IHC in MYC-induced lung tumors. Analogously we observed phospho-Akt1 staining in the CR lung tumors decrease upon *K-ras^G12D^* inactivation (Figure 6B). We observed only minimal changes in the phosphorylation of Akt1 in CM lung tumors upon inactivation of MYC. *K-ras^G12D^*-induced lung tumors exhibited robust conditional Stat3 and Stat5 phosphorylation that was dependent on *K-ras^G12D^* activation (Figure 6C–D). In contrast, MYC-induced tumors had cells that stained highly positive for both phospho-Stat3 and phospho-Stat5, and a large proportion of highly positive phospho-Stat3 and phospho-Stat5 cells remained after MYC inactivation (Figure 6C–D).

**Figure 7:**
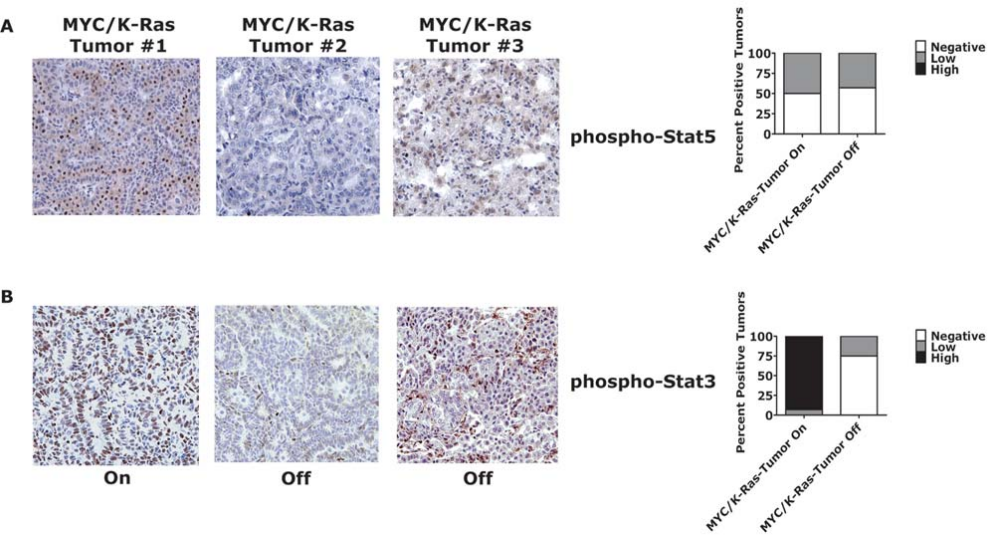
Combined inactivation of MYC and *K-ras^G12D^* in lung tumor cells results in a shutdown of Stat3 signaling. (A) Representative phospho-Stat5 and (B) phospho-Stat3 IHC analysis demonstrates little to no levels of nuclear staining in cells following dual inactivation of MYC/*K-ras^G12D^* (n = 3 “On” & 6 “Off”) similar to conditional *K-ras^G12D^*–induced tumors. MYC/K-ras #2-3 represent independent inactivated tumors with no to highest amount of staining observed, respectively. IHC was performed similar to Figure 1D with stated antibodies with CMR-induced lung tumors that were activated or inactivated (2–11 weeks). Adjacent bar graph panels represent scoring of individual tumors for IHC staining: negative, low (<50% positive cells) or high (≥50% positive cells) for phospho-Stat5 and phospho-Stat3 positive tumors.

Surprisingly, whereas single *K-ras^G12D^*-induced lung tumors exhibited a high degree of Stat5 phosphorylation, dual MYC/*K-ras^G12D^*-induced lung tumors did not (compare Figures 6C and 7A). CMR lung tumors did show a high degree of Stat3 phosphorylation that decreased upon simultaneous inactivation of both MYC/*K-ras^G12D^* in persistent lung tumors (Figure 7B). Thus, *K-ras^G12D^*- or dual MYC/*K-ras^G12D^*-initiated lung tumors demonstrated a decrease in Stat3 phosphorylation upon oncogene inactivation that was associated with reversible tumorigenesis.

**Figure 8:**
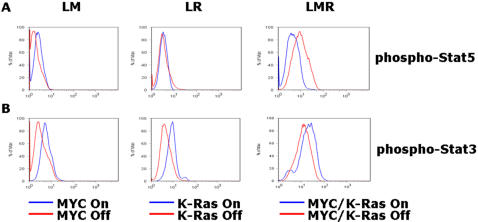
The inactivation of MYC and/or *K-ras^G12D^* in lymphoma is associated with the dephosphorylation of Stat3. Oncogene inactivation in lymphoma demonstrates decrease in Stat3 signaling. (A) LM lymphoma cells show decreased phospho-Stat 5 staining by flow cytometry analysis upon oncogene inactivation, while LR and LMR lymphoma cells do not. (B) LM, LR and LMR lymphoma cells show decreased phospho-Stat 3 staining by flow cytometry analysis following oncogene inactivation.

Next, we examined the consequences of MYC and/or *K-ras^G12D^* inactivation in lymphoma. LM, LR, and LMR lymphoma cells all exhibited phosphorylation of Stat3 that decreased upon inactivation of MYC and/or *K-ras^G12D^* (Figure 8A–B). In contrast, despite the fact that LM, LR, and LMR lymphomas all regress upon MYC and/or *K-ras^G12D^* inactivation, phospho-Stat5 decreased upon MYC, but not *K-ras^G12D^* or dual MYC/*K-ras^G12D^* inactivation. Collectively our results illustrate that for both MYC/*K-ras^G12D^*-induced lung tumors and lymphomas dephosphorylation of Stat 3 is correlated with the ability of oncogene inactivation to induce tumor regression.

## Discussion

Targeting single oncogenes is not likely to be effective in all cases for the treatment of human cancers [38], [51]. Murine models provide a preclinical strategy to identify which combination of oncogenes are most likely to be effective [30], [52]. To our knowledge, our study is the first to examine experimentally using conditional transgenic model systems if the combined inactivation of two oncogenes is more likely to be effective in the treatment of cancer *in situ*. Using our models we interrogate the role of MYC and *K-ras^G12D^* alone or in combination for the initiation and maintenance of lung and hematopoietic tumorigenesis. The inactivation of *K-ras^G12D^* but not MYC could induce complete tumor regression in lung adenocarcinomas; whereas in marked contrast, single *K-ras^G12D^*- or MYC-inactivation both succeeded in inducing sustained regression in lymphomas. However, the combined inactivation of both *K-ras^G12D^* and MYC was capable of inducing complete regression in both lung tumors and lymphomas.

Our data highlight two important considerations in targeted therapeutics: first, initiation of tumorigenesis by a specific oncogene does not mean inactivation of that specific oncogene will be sufficient to induce tumor regression; and second, that the consequences of the inactivation of a particular oncogene are pointedly dependent on tissue context. Specifically, we have demonstrated that the K-Ras pathway and its down-stream effector Stat3 are correlated with the ability of *K-ras^G12D^* or MYC to initiate lung tumorigenesis and that down regulation of the K-Ras/Stat pathway is strongly correlated with lung tumor and lymphoma regression. We conclude that the K-Ras/Stat3 pathways have a dominant role in the initiation and maintenance of lung tumors.

Our experimental model system re-examines the classic experiments first demonstrating the cooperation between *c-myc* and v-Ha*-ras* for malignant transformation *in vivo* [2]. Identical to previous results using conventional transgenic models [2], MYC and *K-ras^G12D^* cooperated to induce tumorigenesis in lymphocytes (compare LM, LR and LMR mice; Figure 3B). In contrast, MYC failed to cooperate with *K-ras^G12D^* to induce lung adenocarcinomas (compare CM, CR and CMR mice; Figure 3A). Thus, whether or not MYC and *K-ras^G12D^* functionally cooperate to activate critical tumor promoting pathways appears to depend upon the specific tissue context. In lung adenocarcinomas induced by MYC and/or *K-ras^G12D^*, tumors exhibited activation of the K-Ras/Stat3 signaling pathway. Apparently, MYC activation is not capable of initiating lung tumorigenesis without activation of the mediators of the K-Ras/Stat3 pathway, perhaps accounting for why MYC does not appear to cooperate with *K-ras^G12D^* to induce lung tumorigenesis. For lung tumorigenesis, there must be an essential role for activation of the K-Ras pathway or downstream mediators such as the Stat pathway. Similarly, the combined inactivation of MYC and *K-ras^G12D^* was now capable of reversing lung tumorigenesis in contrast to MYC-induced lung tumors, because under these circumstances the K-Ras pathway and presumably the downstream Stat3 pathway can be conditionally inactivated.

Notably, our results are highly consistent with several elegant studies that illustrated that mutation of the K-Ras pathway in breast tumorigenesis can reduce the dependence of tumors on sustained MYC overexpression [16], [17], [20]. We acknowledge that the differences seen between tumor initiation and maintenance could be secondary to differences in the expression of MYC and *K-ras* from the two different tissue specific promoters and/or may be confined to the particular genetic background of the mice used in our study. Nevertheless, we speculate that the combined inactivation of both the MYC and K-Ras pathways in these breast tumor models will also result in complete tumor regression.

In our lung tumor model system other genes are likely to be somatically activated in the *EGFR/BRAF/KRAS* pathway or parallel pathways that may also contribute to the escape from the requirement of MYC expression. This is evidenced in our study by the inactivated CM (Figure 6) and CMR (Figure 7) lung tumors that did not demonstrate aberrant signaling in any of the pathways we examined. Possible candidates to undergo such mutations include a multitude of gene products described in studies of human lung tumors [53]–[62], some of these studies have implicated the EGFR/IL-6/Stat3 pathway in the pathogenesis of lung adenocarcinomas [43], [49], [50], [61]–[63]. Stat3 and Stat5 transcription factors have been widely implicated in the pathogenesis of tumors [64], [65] and are known to be downstream targets of K-Ras [27], [44], [48]–[50]. Phosphorylation of Stat3/5 promotes homotetramerization, followed by nuclear translocation and increased transcription of target genes critical for cell growth, survival, and angiogenesis [64], [65]. Persistent activation of Stat3/5 was found in the majority of our inactivated MYC-induced lung tumors, as evidenced by elevated phosphorylation and nuclear localization by IHC (Figure 6C–D). Consistent with a role of Stat3/5 in oncogene-addiction, phosphorylation of Stat3/5 has been shown to diminish in tumor cells undergoing apoptosis upon oncogene inactivation *in vitro* [41], [42]. Phosphorylated Stat3/5 appears to be particularly important for survival of human lung adenocarcinoma cells harboring certain *EGFR* mutations [43], [49], [62]. Similar to the lung cancer mouse models described above, in these *EGFR* mutated lung cancers behave in an oncogene-addicted fashion following treatment with EGFR tyrosine kinase inhibitors [33], [63].

Our observations illustrate that the combined inactivation of multiple oncogenes is more likely to be effective to treat some cancers [30], [38], [51], [52]. The potential of targeting multiple oncogenic pathways in the treatment of human cancer has recently been illustrated in brain tumor cell lines *in vitro* [38]. The identification of the best gene products to therapeutically target in cancers is very likely to be much more complicated than simply identifying the genes mutated in a given tumor, as has recently been illustrated in human lung cancer patients who become resistant to tyrosine kinase inhibitors [37], [51]. In this work, we illustrate that even the knowledge of the oncogene that initiated tumorigenesis is not necessarily sufficient to identify a gene product whose inactivation will result in tumor regression. The generation of transgenic mice with multiple conditional oncogenes is a tractable preclinical platform to define the combination of oncogenic targets most likely to be effective in the treatment of cancer.

## Materials and Methods

### Transgenic mice

The TetO-c-MYC and CCSP-rtTa transgenic lines generated for these experiments was described previously [15]. The Eμ-tTA, and *K-ras4b^G12D^* transgenic lines were kindly provided by H. Bujard (Universität Heidelberg, Germany), and H. Varmus (Memorial Sloan-Kettering Cancer Center, New York), respectively. Mice were mated and screened by PCR as below. MYC and/or *K-ras^G12D^* expression was activated in the CM, CR, and CMR lung lines by administering doxycycline (Sigma) to the drinking water weekly [100 mg/mL] starting at the age of 3-4 weeks. All procedures were performed in accordance with APLAC protocols and animals were housed in a pathogen-free environment.

### Oncogene Inactivation

Lung mice were followed by micro-computed tomography (microCT) scans for a total of 16 weeks. Serial microCT scans were performed at −10, −6, −2, 0, 2 and 6 weeks relative to oncogene inactivation occurring at time point “0”. Oncogenes were inactivated in the CM, CR and CMR cohorts in week 10 by removing doxycycline from the animals' drinking water. Oncogenes were inactivated in the LM, LR and LMR cohorts by injecting mice with 100 µg of doxycycline in PBS IP and adding doxycycline [100 µg/ml] to the drinking water weekly.

### PCR genotyping

DNA was isolated from mouse tails using the Qiaprep DNeasy kit (Qiagen) in accordance with the manufacturer's directions. The CCSP-rtTA segment was detected using the following primers: CCSP-F 5′-ACTGCCCATTGCCCAAACAC-3′ and CCSP-R 5′-AAAATCTTGCCAGCTTTCCCC-3′ (yielding a 440 bp product). The TetO-Myc construct was detected with the following primers: Myc-F 5′-TAGTGAACCGTCAGATCGCCTG-3′ and Myc-R 5′-TTTGATGAAGGTCTCGTCGTCC-3′ (yielding a 450 bp product). TetO-K-ras*^G12D^* and Eμ-SR-tTA werescreened as described previously [23]. DNA was amplified using the following PCR protocol: 94°C denaturation for 2 minutes followed by 35 cycles of 94°C for 15 seconds, 59°C annealing for 30 seconds, and 72°C for 30 seconds, followed by a 5 minute extension at 72°C. PCR products were resolved on a 1.5% gel.

### SYBER-green quantitative RT-PCR and RT-PCR

Total RNA was isolated from tissue using the Strataprep total RNA Miniprep Kit (Stratagene) according to the manufacturer's directions. Samples were treated with RQ1 RNase-Free DNase (Promega) and RT–PCR was performed using Superscript One-Step RT–PCR (Life Technologies) for 35 cycles with an annealing temperature of 57°C with 0.25 µg of total RNA per sample. Control reactions were run using Taq polymerase without RT enzyme (Perkin Elmer). cDNA was generated from 1 µg of total RNA using the Superscript II kit (Invitrogen Technologies). 50 µg of cDNA equivalents were amplified for the transcript described below in an ABI-prism 7700 (Perkin Elmer Applied Biosystems) for 40 cycles using SYBR green PCR Master mix (Perkin Elmer Applied Biosystems) according to manufacturer's directions. PCR reactions were performed in at least triplicate in a final volume of 20 µL. Thermal cycling conditions were: 95°C for 10 minutes, followed by 40 cycles of 95°C for 15 seconds, 57°C for 30 seconds, 72°C for 30 seconds, and a dissociation stage consisting of 95°C for 15 seconds, 60°C for 15 seconds, and 95°C for 15 seconds. Following amplification, the data was processed with the analysis program Sequence Detection Systems v2.2.2 (Applied Biosystems). For each sample, the level of RNA for the genes of interest was standardized to the level of ubiquitin within that sample; subsequently, the level of a transcript of interest was normalized to the expression of that transcript in wildtype lung. Primers for qRT-PCR were the following: transgenic *K-ras* exon 4b (K-ras4b-fwd 5- CAAGGACAAGGTGTACAGTTATGTGACT-3) and downstream primer mp-1 pA (mp-1-real time-rev 5-GGCATCTGCTCCTGCTTTTG-3); endogenous *K-ras4b* 3UTR (K-ras-4b-UTR-fwd 5-GCAGGGTTGGGCCTTACAT-3 and K-ras-4b-UTR rev 5-ATGCGTCGCCACATTGAAT-3); MYC (MYC forward 5′-ACCAGATCCCGGAGTTGGAA-3′) and (MYC reverse 5′-CGTCGTTTCCGCAACAAGTC-3′); ornithine decarboxylase (ODC) (ODC forward 5′-CTGTGCTTCTGCTAGGATCAATGT-3′) and (ODC reverse 5′-GCCTTAACACAAGCTAAACTTGCA-3′); nucleolin (nucleolin forward 5′-GGAGGCCATGGAAGATGGAG-3′) and (nucleolin reverse 5′-CACCTCTGCCTCCGAAACCT-3′); and ubiquitin (ubiquitin forward 5′-AGCCCAGTGTTACCACCAAG-3′) and (ubiquitin reverse 5′-ACCCAAGAACAAGCACAAGG-3).

### Histology and Immunohistochemistry

Tissues were fixed in 10% buffered formalin for 24 h and then transferred to 70% ethanol until embedding in paraffin. Tissue sections 5 µm thick were cut from paraffin embedded blocks, placed on glass slides and hematoxylin and eosin (H&E) staining was performed using standard procedures (Stanford Histology Core). Antibodies used in our study: c-Myc (C19) (Santa Cruz Biotech.), phospho-AKT-S497 (Cell Signaling Tech.), phospho-EGFR-Y1173 (Cell Signaling Tech.), phospho-Erk1/2-T202/Y204 (Cell Signaling Tech.), phospho-Stat3-Y705 (Cell Signaling Tech.) and phospho-Stat5-Y694 (Cell Signaling Tech.). Samples were dewaxed in xylene and rehydrated in a graded series of ethanols. Antigen retrieval for c-Myc, phospho-AKT and phospho-EGFR were performed by 14 min microwave irradiation in citrate-based Antigen Unmasking Solution (Vector Laboratories, Burlingame, CA, USA). Antigen retrieval for phospho-Stat3 and -Stat5 were performed by 14 min microwave irradiation in EDTA, pH 8.0, and antigen retrieval for phospho-Erk1/2 was performed by10 min incubation in Pronase (Roche, Basel, Switzerland). Endogenous peroxidases were blocked in either 3% hydrogen peroxide in deionized water (phospho-AKT, -pErk, -EGFR and -pStat3/5) or 0.3% hydrogen peroxide in methanol (c-Myc) for 10–20 minutes. Non-specific binding was blocked with 5–10% goat serum for 60 minutes. Primary antibodies were used at appropriate dilutions (c-Myc, phospho-AKT, and -pErk at 1∶100; phospho-Stat5 at 1∶200; and phospho-Stat3 and -EGFR at 1∶50) and sections incubated overnight at 4 degrees Celsius. Detection was conducted using the Vector Elite ABC detection kit (Vector Laboratories) with 3,3′-diaminobenzidine tetrahydrochloride as the chromogen. Sections were counterstained with Gill's hematoxylin (Vector Laboratories).

### Western blot analysis

Western analysis was performed using conventional techniques [66]. Tissues were disrupted and protein was isolated using a pestle and tube homogenizer in NP-40 lysis buffer. Equal protein was loaded in each lane, as quantitated by the Bicinchoninic Acid (BCA) Protein Assay (Pierce, Rockford, Illinois, United States). Proteins were electrophoresed on 10% Tris-HCl polyacrylamide gels at 100 V for 60 min and transferred on PVDF membranes at 100 V for 60 min. Blotting was then performed as directed by the antibody manufacturer. MYC protein expression was detected using the 9E10 antibody that recognizes human MYC (Santa Cruz Biotech.).

### Computed Tomography

Micro-computed tomography (µCT) scans were performed on a custom GEHC (London, Ontario) RS150 cone-beam scanner, which uses a fixed anode with tungsten target source. Animals were anesthetized with 2% isofluorane in a nitrogen/oxygen mixture. Scans were performed at 97 µm resolution, using a 70 kV (40 mA) beam to acquire images at 286 radial views over 200 degrees around the subject. Four frames were exposed and averaged in each position. Data were corrected using the GEHC reconstruction utility and volumes generated using the same application, which were viewed using the GEHC Microview software.

### Tumor Volume Measurements

We used the open source application, ITK-Snap, for segmentation of the lung nodules in three-dimensions [67]. The post-processing of the segmented data provides the voxel counts and the volume (cubic millimeters) and displays the shape of the segmented structure. We calculated the volume of individual lung tumor nodules right before and 6 weeks following oncogene-inactivation. Volumes at 6 weeks were normalized relative to the volume before oncogene-inactivation and all values for a given genotype were averaged: CR, n = 11; CM, n = 8; and CMR, n = 10.

### Intracellular phospho-protein detection using flow cytometry

LM, LR, and LMR- lymphoma derived cell lines were treated with doxycyline *in vitro*. 1 million cells from each condition were fixed for 10 minutes in 1.6% paraformaldehyde at 37°C, permeabilized for 10 minutes in 100% methanol at room temperature, washed twice with PBS 1% BSA, then stained with 10 ul anti-Phospho-Stat 5: Alexa-488(BD Biosciences Pharmingen) or 10ul anti-Phospho-Stat 3: Alexa-488 (BD Biosciences Pharmingen) in 100 ul PBS 1% BSA, incubated for 30 minutes in the dark at room temperature. Finally, samples were washed once with PBS 1% BSA and then analyzed using a benchtop FACSCAN (Becton- Dickinson) flow cytometer. 10,000 ungated events were collected per sample and live cells were gated for analysis.

### *K-Ras* mutation analysis

Total genomic DNA was harvested using DNeasy Blood & Tissue Kit (Qiagen) as directed by the manufacturer and PCR amplified using primers specific for *K-Ras*. PCR products were purified with a QIAquick column (Qiagen) as directed by the manufacturer and sequenced to detect point mutations at codons 12, 13, and 61.

### Statistics

Survival graphs were generated by the product limit method of Kaplan and Meier and log-rank analysis was utilized for differences between proportions. Pair-wise and multiple comparisons were made using Mann-Whitney and Kruskal-Wallis nonparametric tests, respectively. Analysis was facilitated using Prism v5.0 by GraphPad.

## Supporting Information

Figure S1 MYC transcriptional targets in the lung tumors. The canonical MYC transcriptional targets (A) ornithine decarboxylase (ODC) and (B) nucleolin were assayed by qRT-PCR as performed in Figure 1B. This pattern of MYC target expression supports the conditional and lung specific regulation of a functional MYC gene product in the bitransgenic CM mice.
(5.37 MB TIF)

Figure S2 Lung specific co-expression of MYC and K-rasG12D transgenes in MYC/K-rasG12D mice. Genotype and lung specific expression of (A) MYC and (B) K-rasG12D transgenes were assayed by RT-PCR using cDNA templates generated from mRNA extracted from lung tumors of CM, CR and CMR mice. Minus reverse transcriptase (-RT) and water (H20) negative controls were performed concurrently. The -RT control shown was generated using the CMR mRNA. (C) Ubiquitin control RT-PCR.
(3.33 MB DOC)

Figure S3 MYC-induced lung tumors do not contain dominant mutations in K-Ras. (A) Schematic of primers used to amplify genomic DNA followed by sequencing of PCR products for K-Ras and an example using CR mice tissue as a positive control. (B) Two primary tumors and one derived cell line transplanted into SCID mice were assayed for mutations in exon one hotspots, codon 12 and 13. (C) Similar samples assayed for exon 2 hotspot codon 61.
(9.43 MB TIF)
