# Supplementary figures and images for: Combined Inactivation of MYC and K-Ras Oncogenes Reverses Tumorigenesis in Lung Adenocarcinomas and Lymphomas
Source: PLoS One. 2008 May 7;3(5):e2125. doi: 10.1371/journal.pone.0002125 (PMC2365560; doi:10.1371/journal.pone.0002125)

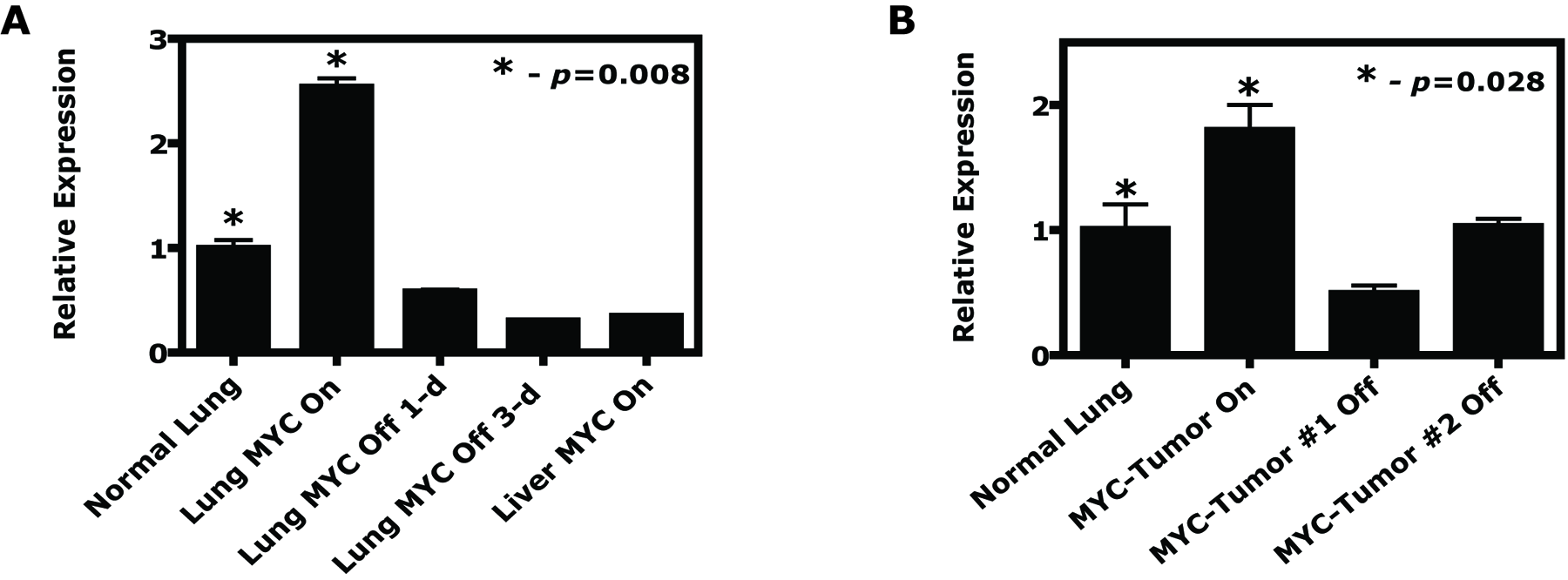

Supplement: Figure S1 — MYC transcriptional targets in the lung tumors. The canonical MYC transcriptional targets (A) ornithine decarboxylase (ODC) and (B) nucleolin were assayed by qRT-PCR as performed in Figure 1B. This pattern of MYC target expression supports the conditional and lung specific regulation of a functional MYC gene product in the bitransgenic CM mice. (5.37 MB TIF) [file pone.0002125.s001.tif]

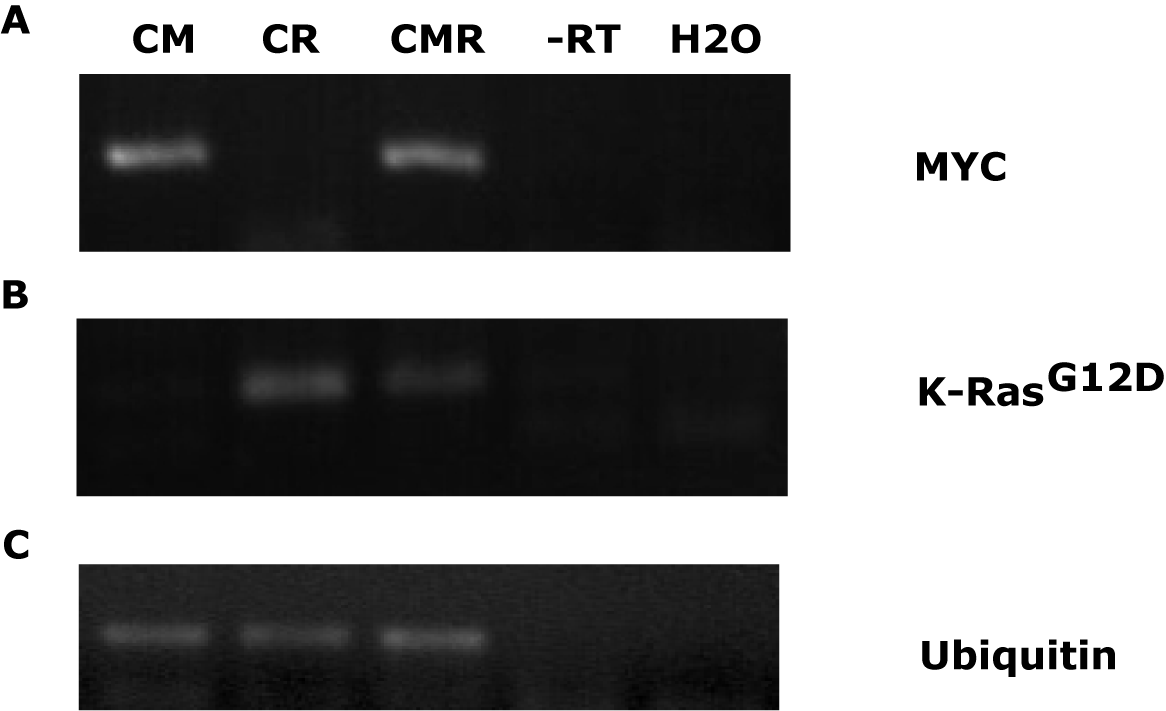

Supplement: Figure S2 — Lung specific co-expression of MYC and K-rasG12D transgenes in MYC/K-rasG12D mice. Genotype and lung specific expression of (A) MYC and (B) K-rasG12D transgenes were assayed by RT-PCR using cDNA templates generated from mRNA extracted from lung tumors of CM, CR and CMR mice. Minus reverse transcriptase (-RT) and water (H20) negative controls were performed concurrently. The -RT control shown was generated using the CMR mRNA. (C) Ubiquitin control RT-PCR. (3.33 MB DOC) [file pone.0002125.s002.tif]

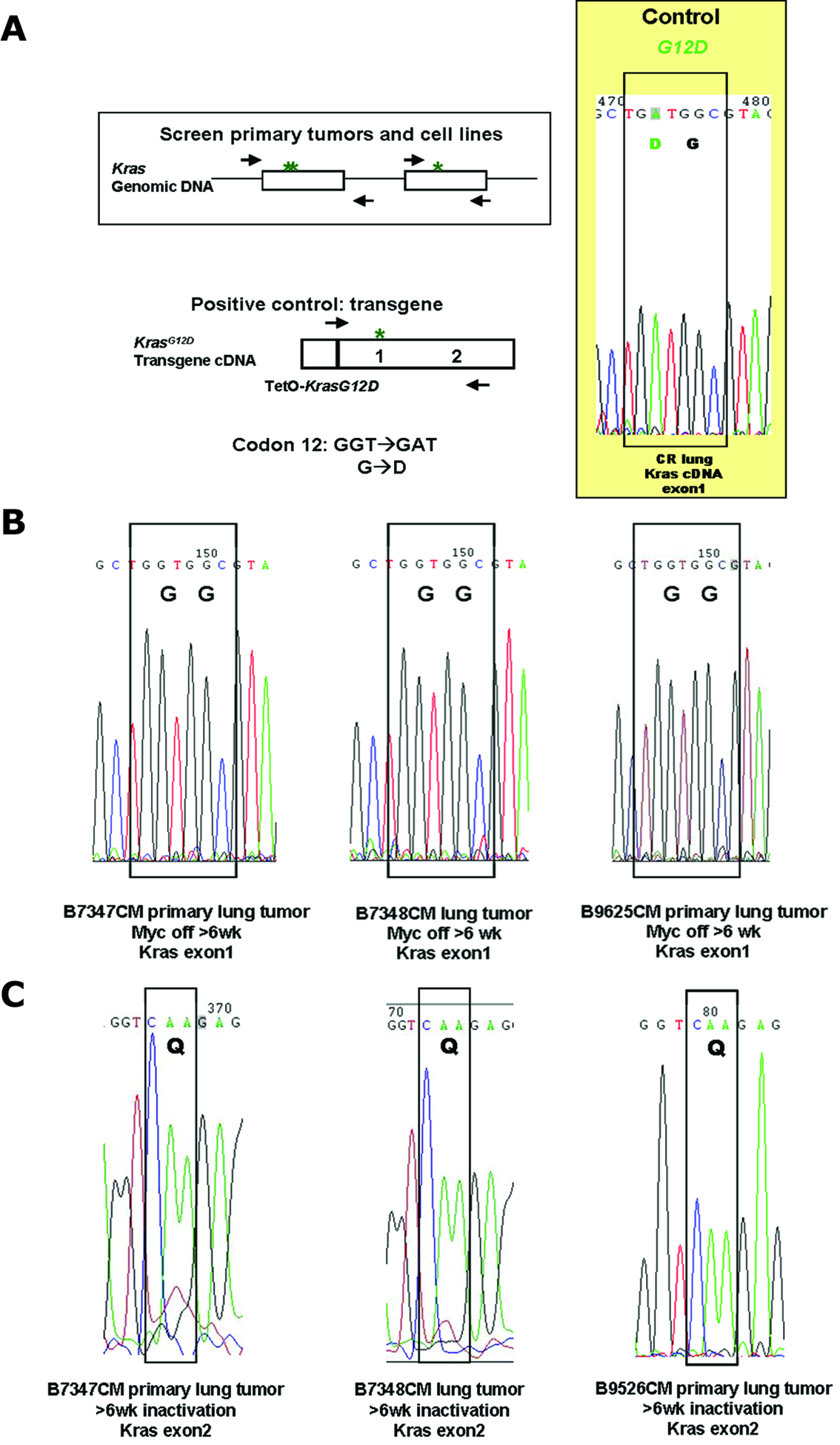

Supplement: Figure S3 — MYC-induced lung tumors do not contain dominant mutations in K-Ras. (A) Schematic of primers used to amplify genomic DNA followed by sequencing of PCR products for K-Ras and an example using CR mice tissue as a positive control. (B) Two primary tumors and one derived cell line transplanted into SCID mice were assayed for mutations in exon one hotspots, codon 12 and 13. (C) Similar samples assayed for exon 2 hotspot codon 61. (9.43 MB TIF) [file pone.0002125.s003.tif]
